# Supplementary figures and images for: Temporal expression profiles of lncRNA and mRNA in human embryonic stem cell-derived motor neurons during differentiation
Source: PeerJ. 2020 Nov 13;8:e10075. doi: 10.7717/peerj.10075 (PMC7668206; doi:10.7717/peerj.10075)

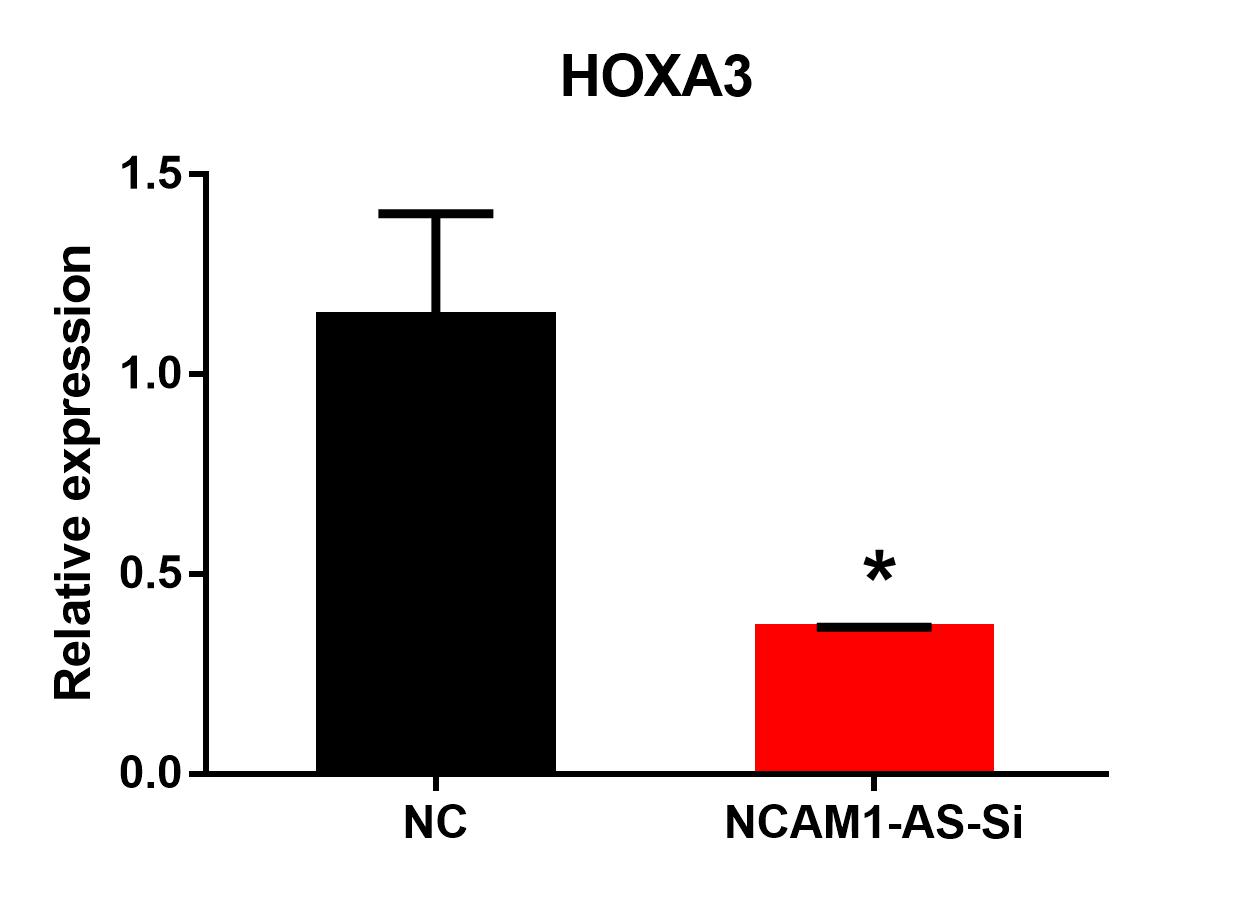

Supplement: Figure S1 — NC, NC Smart Silencer; NCAM1-AS-Si, NCAM1-AS Smart Silencer. * P < 0.05. [file peerj-08-10075-s006.jpg]
